# The Tumor Suppressive mir-148a Is Epigenetically Inactivated in Classical Hodgkin Lymphoma

**DOI:** 10.3390/cells9102292

**Published:** 2020-10-14

**Authors:** Julia Paczkowska, Joanna Janiszewska, Julia Bein, Markus Schneider, Kinga Bednarek, Adam Ustaszewski, Sylvia Hartmann, Martin-Leo Hansmann, Maciej Giefing

**Affiliations:** 1 Institute of Human Genetics, Polish Academy of Sciences, 60-479 Poznan, Poland; julia.paczkowska@igcz.poznan.pl (J.P.); joanna.janiszewska@igcz.poznan.pl (J.J.); kinga.bednarek@igcz.poznan.pl (K.B.); adam.ustaszewski@igcz.poznan.pl (A.U.); 2 Dr. Senckenberg Institute of Pathology, Goethe University Hospital, 60590 Frankfurt am Main, Germany; julia.bein@kgu.de (J.B.); markus.schneider@uk-essen.de (M.S.); s.hartmann@em.uni-frankfurt.de (S.H.); martin-leo.hansmann@kgu.de (M.-L.H.); 3 Reference and Consultant Center for Lymph Node and Lymphoma Pathology, Goethe University, 60590 Frankfurt am Main, Germany; 4 Frankfurt Institute for Advanced Studies, 60438 Frankfurt am Main, Germany

**Keywords:** cHL, epigenetic, microRNA, DNA methylation, mir-148a

## Abstract

DNA methylation was shown previously to be a crucial mechanism responsible for transcriptional deregulation in the pathogenesis of classical Hodgkin lymphoma (cHL). To identify epigenetically inactivated miRNAs in cHL, we have analyzed the set of miRNAs downregulated in cHL cell lines using bisulfite pyrosequencing. We focused on miRNAs with promoter regions located within or <1000 bp from a CpG island. Most promising candidate miRNAs were further studied in primary Hodgkin and Reed-Sternberg (HRS) cells obtained by laser capture microdissection. Last, to evaluate the function of identified miRNAs, we performed a luciferase reporter assay to confirm miRNA: mRNA interactions and therefore established cHL cell lines with stable overexpression of selected miRNAs for proliferation tests. We found a significant reverse correlation between DNA methylation and expression levels of mir-339-3p, mir-148a-3p, mir-148a-5p and mir-193a-5 demonstrating epigenetic regulation of these miRNAs in cHL cell lines. Moreover, we demonstrated direct interaction between miR-148a-3p and *IL15* and *HOMER1* transcripts as well as between mir-148a-5p and *SUB1* and *SERPINH1* transcripts. Furthermore, mir-148a overexpression resulted in reduced cell proliferation in the KM-H2 cell line. In summary, we report that mir-148a is a novel tumor suppressor inactivated in cHL and that epigenetic silencing of miRNAs is a common phenomenon in cHL.

## 1. Introduction

DNA methylation is a crucial mechanism responsible for deregulation of gene expression in human neoplasms. Both global DNA hypomethylation and hypermethylation of CpG islands, located in gene promoter regions, were widely described in tumorigenesis. Global hypomethylation increases genomic instability, whereas promoters hypermethylation result in the silencing of gene expressions [1]. However, recently new insight into the mechanisms of gene expression regulation by DNA methylation was provided. As shown for MMP-9 genes in melanoma cell lines, intragenic hypermethylation, in contrast to hypermethylation of promoter regions, positively correlates with gene expression level [2].

The exceptional importance of aberrant DNA methylation in the development of classical Hodgkin lymphoma (cHL) was demonstrated by several studies showing that DNA hypermethylation attenuates the expression of genes responsible for normal B-cell development [3,4]. Consequently, the neoplastic Hodgkin and Reed–Sternberg cells (HRS) of cHL show a characteristic loss of the B-cell phenotype and an increased immune escape potential [5]. Concerning the important role of DNA methylation in cHL pathogenesis, we propose that DNA methylation is co-responsible for deregulation of miRNA expression in cHL in a similar manner to protein coding genes.

The phenomenon of miRNA silencing by DNA methylation in cancer is gaining attention recently. It is known that mir-155, mir-152, mir-137, mir-31 and mir-874 expression are regulated by DNA methylation in solid tumors such as breast and prostate cancer [6,7]. Similarly, it has been shown that this mechanism contributes to miRNA downregulation in hematological malignances such as infant acute lymphoblastic leukemia or mantle cell lymphoma [8,9]. However, the significance of this process for cHL pathogenesis remains unknown and the available literature data on this phenomenon are scarce. Among the few published studies, miRNA promoter methylation and subsequent changes in microRNA expression after 5-aza-2-deoxycytidine (5-Aza-dC) treatment were described by Navarro et al. in two cHL cell lines (L-428 and L-1236) [10]. The authors have shown that altogether the expression of 13 microRNAs were induced after global DNA demethylation in both cell lines, suggesting their epigenetic inactivation.

Intrigued by these findings, we aimed to identify epigenetically inactivated miRNAs within the group of 23 downregulated miRNAs in cHL which might act as potential tumor suppressors in the disease. We decided to use unmodified cHL and non-Hodgkin lymphoma (NHL) cells lines as well as normal germinal center B-cells (GCB) from non-tumor donors in contrast to a previous study [10], to assess baseline methylation states in these malignant and normal B cells.

As a result of our analysis, we identified an epigenetically regulated microRNA mir-148a, not previously reported for cHL which could play an important role in cHL pathogenesis since it is known to be involved in the regulation of B-cell differentiation and of germinal center transcription factors [11].

## 2. Materials and Methods

### 2.1. cHL and NHL Cell Lines

Seven cHL cell lines (L-428, HDLM-2, KM-H2, L-1236, U-HO1, SUP-HD1, L-540) and 10 NHL cell lines (RAJI, DAUDI, RAMOS, NAMALWA, CA-46, VAL, OCI-LY1, OCI-LY3, OCI-LY7, SU-DHL-6) were obtained from Deutsche Sammlung von Mikroorganismen und Zellkulturen GmbH (DSMZ) or were kindly provided by Andreas Bräuninger (University Hospital Giessen, Germany) (cells: L-428, KM-H2, L-1236, L-540) [12,13]. Detailed information on cell culture conditions are presented in Table S1. Cell lines were authenticated by STR DNA profiling.

### 2.2. Laser Capture Microdissection (LCM) of HRS and Non-Tumor Cells

Frozen lymph nodes of 14 patients with cHL (10 cases: 4 nodular sclerosis, 3 mixed cellularity, 2 lymphocyte-rich and 1 undefined subset used for miRNA expression analysis and 6 cases: 3 nodular sclerosis, 2 mixed cellularity and 1 lymphocyte-rich used for DNA methylation analysis) were obtained from Dr. Senckenberg Institute of Pathology, Goethe University Hospital, Frankfurt am Main, Germany. Detailed information about clinical samples are presented in Table S2. Frozen sections (5–10 μm) of the lymph nodes were mounted on membrane-covered slides (PALM, Zeiss, Bernried, Germany) and fixed in acetone. HRS cells were microdissected immediately after H&E or CD30 immunostaining. For miRNA expression analysis approximately 1000 HE stained cells per case were collected onto adhesive caps. For DNA methylation analysis, anti-CD30 (Clone BerH2, DAKO, Glostrup, Denmark, Super Sensitive Link-Label IHC Detection System BIOGENEX, San Ramon, CA, USA) pretreated slides were used to dissect 2 × 200 HRS cells and 2 × 200 non-tumor cells per case into 20 μL PCR buffer without MgCl_2_ (Expand High Fidelity, Roche, Grenzach, Germany) supplemented with 0.1% Triton X-100. LCM was performed using the PALM laser capture microdissection microscope/system (PALM MicroBeam, Zeiss, Bernried, Germany). The study was approved by the local ethics committee of the Goethe University Hospital (157/17 from 06.04.2017).

### 2.3. Sorting of GCB CD77^+^ Cells

CD77^+^ GCB cells were purified from fresh tonsils obtained from tonsillectomies of chronic hyperplastic tonsillitis using magnetic activated cell sorting (MACS; Miltenyi Biotech, Bergisch Gladbach, Germany), as described previously [14]. Informed consent was obtained from all patients according to the declaration of Helsinki. The study was approved by the local ethics committee of Goethe University Hospital (157/17 from 06.04.2017).

### 2.4. DNA Isolation

DNA isolation from cell lines was performed by phenol/chlorophorm extraction with the use of Phase Lock Gel™ tubes (5Prime Quantabio, Beverly, MA, USA) and ethanol precipitation and for MACS sorted CD77^+^ GCB cells by using a DNeasy Blood and Tissue Kit (Qiagen, Hilden, Germany). DNA from LCMed HRS and bystander cells for methylation analysis were obtained by cell lysis in Tris-Protein K buffer by shaking (600 rpm) in 55 °C for 72 h.

### 2.5. RNA and miRNA Isolation

Total RNA from cell lines was isolated with use of Trizol reagent based on the Chomczynski method [15]. miRNA from sorted GCB and microdissected HRS cells was isolated using miRNeasy Mini Kit (Qiagen).

### 2.6. Real-Time qPCR

#### 2.6.1. miRNA Expression Analysis

The total RNA (10 ng) from cell lines and sorted GCB, and 10 μL of the miRNA containing eluate from HRS cells were transcribed to cDNA with TaqMan™ Advanced miRNA cDNA Synthesis Kit (Applied Biosystem, Foster City, CA, USA) according to the protocol provided by the manufacturer. For reverse transcription, 3’ poly-A tailing and 5’ adaptor sequence ligation were performed and all mature miRNAs were reverse transcribed using RT primers binding to universal sequences present on both the 5′ and 3′ extended ends. Afterward, cDNA was amplified using the Universal miR-Amp Primers and miR-Amp Master Mix. Expression of miR-148a-3p and miR-148a-5p was assessed with TaqMan™ Advanced miRNA Assays (Assay ID 477814_mir and 478718_mir) and normalized to control microRNAs: miR-361-5p and let-7g-5p (Assay ID 478056_mir and 478580_mir). The PCR reaction mix contained: 10 μL 2 × Fast Advanced Master Mix, 1 μL TaqMan^®^ Advanced miRNA Assay, 5 μL of diluted cDNA template (1:10), and 4 μL H_2_O. Reactions were run in triplicate under the following condition: 95 °C for 20 s × 1; (95 °C for 1 s, 60 °C for 30 s) × 40.

#### 2.6.2. Gene Expression Analysis

Total RNA (500 ng) from the cell lines were reverse transcribed into cDNA using the Maxima First Strand cDNA Synthesis Kit (Thermo Fisher Scientific, Waltham, MA, USA). The expression level of putative target genes for miR-148a-3p and for miR-148a-5p was evaluated in reference to the expression of ACTB and GAPDH genes. Primer sequences were designed using the Primer-BLAST software (Primer3 and BLAST) (https://www.ncbi.nlm.nih.gov/tools/primer-blast) (Table S3). The PCR reaction mix for the analyzed genes and GAPDH contained: 2 µL HOT FIREPol EvaGreen qPCR Mix Plus (no Rox) (Solis Biodyne, Tartu, Estonia), 0.2 µL F and R primers (20 pmoμ/µL each), 1 µL cDNA, and 6.6 µL H_2_O. Reactions were run in triplicate under the following condition: 95 °C for 15 min × 1; (95 °C for 15 s, T_A_ °C for 10 s, 72 °C for 15 s) × 40; 1 × 95 °C for 30 s; 1 × 50 °C for 30 s (specific annealing temperature (T_A_) temperatures for all reactions are shown in Table S3). The PCR reaction mix for the ACTB probe consisted of: 5 µL SsoAdvanced Universal Probes Supermix (Bio-Rad, Hercules, CA, USA), 0.5 µL ACTB probe (Bio-Rad, Hercules, USA), 1 µL cDNA, 3.5 µL H_2_O, and reactions were performed in triplicate under the following conditions: 95 °C for 2 min × 1; (95 °C for 5 s, 60 °C for 30 s) × 40.

Real-time qPCR was performed using the CFX96Touch Real-Time PCR System (Bio-Rad, Hercules, USA) according to standard procedures. Results were analyzed using the Gene Expression MacroTM 1.10 software (Bio-Rad).

### 2.7. mir-148a Mutation Screening

Primer sequences for mir-148a amplification were designed using the Primer-BLAST software (https://www.ncbi.nlm.nih.gov/tools/primer-blast) (Table S3). The assay for mutation screening enclosed the genomic sequence of mir-148a with flanking regions (amplified sequence: chr7:25,989,422-25,989,721 GRCh37/hg19). The PCR reaction mix consisted of: 2 µL Colorless GoTaq Flexi Buffer (Promega, Madison, WI, USA), 0.4 µL MgCl_2_ Solution (Promega), 0.3 µL dNTP 10 mM each (Thermo Fisher Scientific), 0.2 µL F and R primers (20 pmol/µL each), 0.25 GoTaq G2 Hot Start Polymerase 5 u/µL (Promega), and 5.65 µL H_2_O and 1 µL DNA template (50 ng/µL). PCR products were obtained using the following conditions: 95 °C for 2 min × 1; (95 °C for 30 s, 55 °C for 30 s, 72 °C for 30 s) × 30, 72 °C for 5 min × 1. PCR products were visualized under UV light (BioDoc-it Imaging System, UVP, Upland, USA) on 1.5% agarose gels stained with SimplySafe (EuRx, Gdansk, Poland) and purified with ExoSap IT (Affymetrix, Santa Clara, USA). DNA sequencing reactions were performed using BigDye^®^ Terminator v3.1 Cycle Sequencing Kit (Applied Biosystems) under the following conditions: 95 °C for 2 min × 1; (96 °C for 10 s, 55 °C for 5 s, 60 °C for 240 s) × 26.

Products were purified by ethanol precipitation, separated using the ABI310 sequencer (Applied Biosystem), analyzed using the Sequencing Analysis (ABI) software, and visualized using Codon Code Aligner v.6.0.2.

### 2.8. Bisulfite Pyrosequencing

DNA bisulfite conversion was performed using EZ DNA Methylation–Gold™ Kit (Zymo Research, Irvine, CA, USA) for cell lines and GCB cells, whereas the EpiTect Plus Bisulfite Kit (Qiagen) was used for microdissected cells. The assays for bisulfite sequencing of miRNA promoter regions were designed using the PyroMark Assay Design Software 2.0.1.15 (Qiagen) (Table S3). For PCR, the PyroMark PCR kit was used to prepare the reaction mixture that contained: 12.5 µL PyroMark Master Mix; 2.5 µL CoralLoad; 0.5 µL of F and R primer (20 pmol/µL), 1 µL of converted DNA (25 ng/1 µL), and 8 µL H_2_O. PCR was performed under the following conditions: 95 °C for 15 min × 1; (94 °C for 30 s, T_A_ °C for 30 s, 72 °C for 30 s) × 45; 72 °C for 10 min × 1; 4 °C ∞. PCR products were visualized on 1.5% agarose gel stained with SimplySafe (EuRx) under UV light (BioDoc-it Imaging System, UVP).

Pyrosequencing was performed using the PyroMark Q24 (Qiagen) sequencer as described previously [16]. Each run included fully methylated (M-commercially available methylated DNA, Millipore, Hilden, Germany) and unmethylated controls (UMET-whole genome amplified DNA from pooled peripheral blood lymphocytes by using GenomePlex Complete Whole Genome Amplification (WGA) Kit, Sigma-Aldrich, Saint Louis, MO, USA). DNA methylation level was assessed as a mean result for all analyzed CpG dinucleotides for each assay. Detailed information about sequences analyzed by pyrosequencing is shown in Table S4.

For mir-148a promoter DNA methylation in microdissected cells, the same PCR conditions as described above were used, however two rounds of PCR were performed as described previously [17]. The first PCR round included: 25 µL PyroMark Master Mix; 5 µL CoralLoad; 1 µL of F and R primer (20 pmol/µL) and 18 µL of converted DNA (whole lysate). The second PCR round included a standard PCR reaction mix with 1 µL of PCR product from the first round as a DNA template.

### 2.9. Dual-Luciferase Reporter Assay

Wild type (WT) or mutant (MUT) miRNA binding sites located in the 3′UTRs of selected genes were cloned into the pmirGLO Dual-Luciferase miRNA Target Expression Vector (Promega) followed by JM109 competent cells transformation (Promega). Vectors were purified using PhasePrep BAC DNA Kit (Sigma-Aldrich) and controlled by Sanger sequencing. WT and MUT oligonucleotides were designed as proposed by Mets et al. and purchased from Genomed company (Warsaw/Poland) (Table S5) [18]. In detail, WT constructs represent miRNA binding sites flanked with 30 +/− bp of the respective 3′UTR. For MUT oligonucleotides, point mutations in miRNA binding sites were introduced in an attempt to abolish the putative interaction between the miRNA and the 3′UTR.

The validation of the miRNA-3′UTR interactions was performed in the HEK 293T cells in two independent transfections and in three technical repetitions using jetPRIME DNA/siRNA (Polyplus-transfection SA, Illkirch-Graffenstaden, France) reagent as follows:

- In total, 500 ng of vector containing the 3′UTR WT sequence + 50 µM of the analyzed miRNA mimic (mirVana^®^ miRNA mimic, MC10263, MC12683, Invitrogen, Carlsbad, CA, USA)
- In total, 500 ng of vector containing the 3′UTR WT sequence + 50 µM of the mimic negative control (mirVana™ miRNA Mimic, Negative Control #1, Invitrogen)
- In total, 500 ng of vector containing the 3′UTR MUT sequence + 50 µM of the analyzed miRNA mimic (mirVana^®^ miRNA mimic, MC10263, MC12683, Invitrogen)
- In total, 500 ng of vector containing the 3′UTR MUT sequence + 50 µM of the mimic negative control (mirVana™ miRNA Mimic, Negative Control #1, Invitrogen)

Cells were lysed with Dual-Glo Luciferase Assay System 24 h after transfection and the bioluminescence signal of firefly luciferase was measured using the GloMax^®^ 96 Microplate Luminometer in reference to internal control of Renilla luciferase.

### 2.10. miRNA Overexpression

The mir-148a insert containing the 3p and 5p miRNA was prepared by PCR amplification using primers specific for the genomic sequence harboring the pre-miRNA-148a hairpin, including approximately 100–250 nt flanking sequence on each site, as described previously [19] (for primer sequences see Table S3). The PCR product with cohesive ends was directly cloned into the pCDH-CMV-MCS-EF1α-GreenPuro vector (SBI, Palo Alto, USA) and used for functional studies. The vector was packaged into lentiviral particles in HEK 293T cells using jetPRIME transfection reagent (Polyplus-transfection SA, Illkirch-Graffenstaden, France). The lentiviral particles were harvested 48 h after transfection and three cHL cell lines (KM-H2, L-1236 and L-540) were independently transduced by the vector carrying the mir-148a (3p and 5p) sequence as well as by the empty vector. The vectors used contain a GFP gene and express the GFP-puromycin resistance fusion gene that enables drug selection of target cells stably expressing the microRNA. After 14 days of antibiotic selection, transduction efficiency was analyzed by flow cytometry and the overexpression of the respective miRNAs was confirmed by real-time qPCR TaqMan^®^ Advanced miRNA Assays. Cultures showing > 75% of transduced cells were used for proliferation assays.

### 2.11. Proliferation Tests

Three transduced cHL cell lines (KM-H2, L-1236 and L-540) were seeded (500,000 cells/per well) in 24-well plates in antibiotic depleted medium after puromycin selection. The CCK8 test (Cell Counting Kit-8, Sigma-Aldrich) was performed in a time dependent manner from days 0 to 8 (measurement was performed every 2 days) to observe the differences between cells transduced with mir-148a expression and the empty vector. CCK8 assay, which is based on bio reduction of WST-8 (2-(2-methoxy-4-nitrophenyl)-3-(4-nitrophenyl)-5-(2,4-disulfophenyl)-2H-tetrazolium, monosodium salt) into formazan by cellular dehydrogenases, was performed to check the influence of miR-148a on cell proliferation. Cells were incubated with CCK-8 for 2 h in 37 °C and the absorbance at 450 nm and 600 nm was measured using the GloMax^®^ 96 Microplate Luminometer. Experiments were performed in 4 replications in three independent reactions. 

Cell proliferation was also analyzed via DNA synthesis measurement using the Click-iT Plus EdU Alexa Fluor 647 Flow Cytometry Assay Kit (Invitrogen). Cells (500,000 cells/per well) were incubated with EdU (5-ethynyl-2′-deoxyuridine) or DMSO as a control for 4 h, fixed and permeabilized with saponin. The fluorescence signal of Alexa Fluor™ 647 Click-iT™ was detected by FlowSight^®^ Imaging Flow Cytometer (Luminex). The experiment was performed in triplicate in two time points (days 0 and 3).

## 3. Results

### 3.1. miRNA Expression in cHL Is Deregulated by DNA Hypermethylation

**Figure 1:**
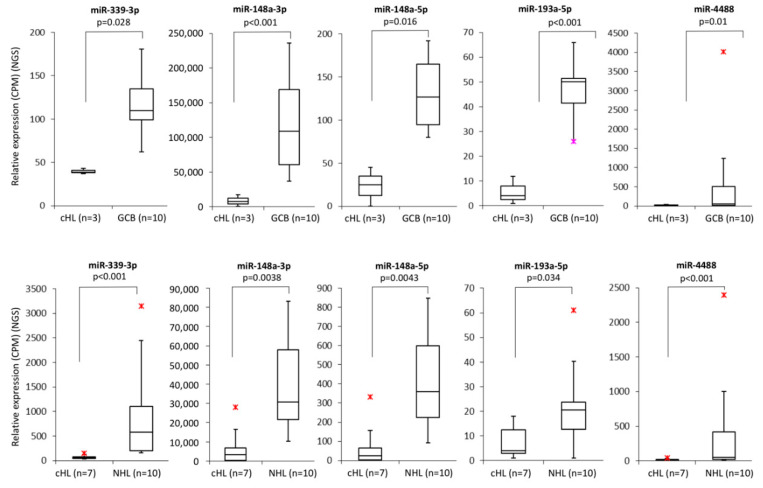
Expression of miRNAs (mir-339-3p, mir-148a-3p, mir-148a-5p, mir-193a-5p and mir-4488) which promoter regions are located within or up to 1000 bp upstream from a CpG island, downregulated in cHL cell lines (*n* = 7) in comparison to NHL cell lines (*n* = 10) (based on NGS sequencing, *p* < 0.05, upper panel). Expression of miRNAs (mir-339-3p, mir-148a-3p, mir-148a-5p, mir-193a-5p and mir-4488) which promoter regions are located within or up to 1000 bp upstream from a CpG island, downregulated in cHL cell lines (*n* = 3) in comparison to sorted GCB 77^+^ from tonsillectomy specimens of chronic hyperplastic tonsillitis (*n* = 10) (based on NGS sequencing, *p* < 0.05, lower panel); 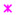/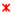-min. and max. outliers.

**Figure 2:**
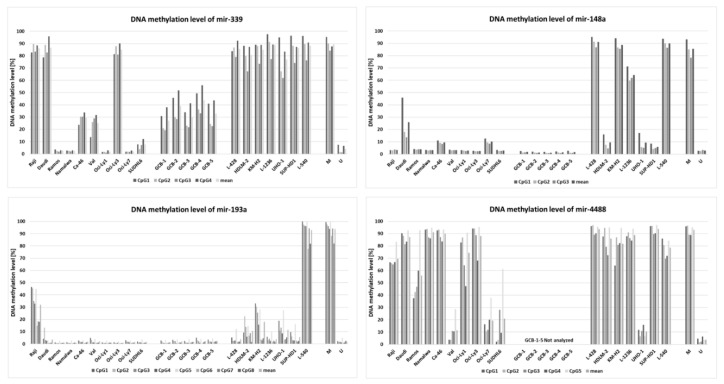
DNA methylation level of promoter regions of downregulated miRNAs (mir-339, mir-148a, mir-193a and mir-4488) in cHL cell lines (*n* = 7), NHL cell lines (*n* = 10) and GCB 77^+^ from tonsillectomy specimens of chronic hyperplastic tonsillitis (*n* = 5) (except mir-4488) (analyzed by DNA bisulfite pyrosequencing).

Within the group of 23 miRNAs found downregulated in cHL in our parallel study (manuscript in preparation), we identified five with promoter regions located within or <1000 bp from a CpG island: miR-339-3p, miR-148a-3p, miR-148a-5p, miR-193a-5p, miR-4488 (Figure 1). To determine whether those miRNAs were regulated by DNA methylation, we have performed bisulfite DNA pyrosequencing for the respective promoter regions of these miRNAs in cHL cell lines (*n* = 7) and NHL cell lines (*n* = 10) as controls. We have found that the promoter region of mir-339 was hypermethylated in all cHL cell lines (range 77–89%) and in 3 of 10 NHL cell lines (range 85–87%), mir-148a in L-428, KM-H2, L-1236 and L-540 cell lines (range 64–91%) and mir-193a only in L-540 (93%). Mir-4488 was hypermethylated in 6 of 7 cHL cell lines (range 78–94%) but also in 5 of 10 NHL cell lines (range 74–91%) (Figure 2). For 3 of 4 analyzed miRNA promoter regions, in the case of miR-339-3p (r = −0.65, *p* < 0.01), miR-148a-3p (r = −0.72, *p* < 0.01), miR-148a-5p (r = −0.74, *p* < 0.01) and miR-193a-5p (r = −0.67, *p* < 0.01), their expression (based on small RNA-seq) inversely correlated with DNA methylation level (Spearman correlation).

Importantly, by further testing of these three regions (promoter of mir-339, mir-148a, mir-193a) in GCB cell pools, we observed no DNA hypermethylation for any of the chosen miRNAs (elevated DNA methylation was observed for mir-339) suggesting that DNA hypermethylation in these regions is a unique characteristic of the neoplastic cells. Because two miRNAs, namely miR-148a-3p and miR-148a-5p, were found to be recurrently silenced by DNA methylation exclusively in 4/7 cHL cell lines and not in any of the tested NHL cell lines or in GCB cells, we focused on these miRNAs in the further analysis.

**Figure 3:**
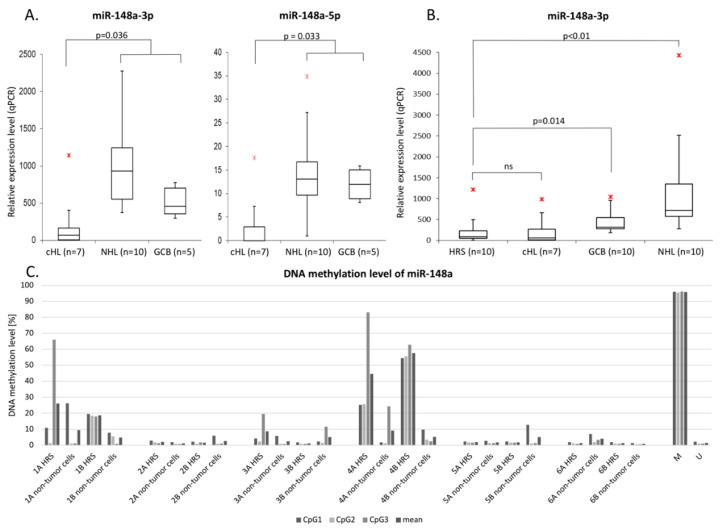
(**A**): Validation of mir-148a-3p/5p downregulation by real-time qPCR in cHL cell lines (*n* = 7) in comparison to NHL cell lines (*n* = 10) and GCB 77^+^ cell pools from tonsillectomy specimens of chronic hyperplastic tonsillitis (*n* = 5) (*p* < 0.05); 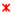
- max. outlier. (**B**): miR-148a-3p downregulation in microdissected HRS cells from cHL cases (*n* = 10) in comparison to cHL cell lines (*n* = 7), NHL cell lines (*n* = 10) and GCB 77^+^ cell pools from tonsillectomy specimens of chronic hyperplastic tonsillitis (*n* = 10) (*p* < 0.05); 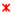
- max. outlier. (**C**): Elevated DNA methylation in primary microdissected HRS cells (case 1 and 4) from cHL cases (*n* = 6) in comparison to non-tumor cells from the same patients.

Lastly, we have confirmed the downregulation of miR-148a-3p and miR-148a-5p in cHL cell lines and GCB cells using real-time qPCR with Taqman probes (Figure 3A). This shows that DNA hypermethylation downregulates miRNA gene expression and contributes to cHL-associated attenuation of miR-148a-3p and miR-148a-5p.

### 3.2. Canonical Gene Inactivation Mechanisms Seldomly Target mir-148a in cHL

In order to identify further mechanisms underlying the deregulation of mir-148a in cHL, we have screened for putative copy number losses by using available results of SNP array platforms for cHL cell lines [20,21]. In two of seven evaluated cHL cell lines (L-1236, HDLM-2) with low (9%) or moderate (64%) mir-148a DNA methylation levels, we found heterozygous deletions that may partially explain the observed downregulation of this miRNA. In addition, we have used Sanger sequencing to identify putative mir-148a loss of function mutations. No genomic variants have been detected in the seven cHL cell lines which strengthens the hypothesis that DNA hypermethylation is the main mechanism of mir-148a deregulation.

### 3.3. mir148a Is Transcriptionally Deregulated and Hypermethylated also in Primary HRS Cells

In order to elucidate if the DNA hypermethylation and downregulation of mir-148a is not only limited to cell lines, we have performed real-time qPCR with Taqman probes in pooled HRS cells from 10 cHL cases. Similarly to what we observed in cHL cell lines, the expression level of miR-148a-3p was significantly lower in primary HRS cells in comparison to NHL cell lines and GCB cells sorted from tonsillectomy specimens of chronic hyperplastic tonsillitis (*p* < 0.05) (Figure 3B). MiR-148a-5p expression in HRS cells was not possible to analyze due to low input of miRNA after microdissection and significantly lower endogenous expression of this miRNA in comparison to miR-148a-3p.

Lastly, we have also confirmed higher mir-148a promoter region DNA methylation levels in microdissected HRS cells from a subset of cHL primary cases by bisulfite DNA pyrosequencing. Two out of six evaluated cases showed elevated methylation as compared to non-tumor bystander cells from the same patient. (Figure 3C).

### 3.4. IL15, HOMER1, SERPINH1 and SUB1 Are mir-148a Target Genes

As we have shown that mir-148a (3p and 5p) downregulation is characteristic of cHL, we were interested in identifying putative target genes that were overexpressed as a consequence of the diminished interaction with these miRNAs. We performed an in silico screen of putative target genes of these miRNAs using miRWalk 2.0 (http://zmf.umm.uni-heidelberg.de/apps/zmf/mirwalk2/index.html). We found 1421 putative target genes for miR-148a-3p, and 613 for miR-148a-5p indicated by their presence in at least 7 of 12 databases in miRWalk. By combining these results with published gene expression microarray data (Affymetryx U95) [22], we have identified 90 candidate genes for miR-148a-3p and 60 for miR-148a-5p, with at least 2-fold increased expression in cHL cell lines (*n* = 4: L-428, L-1236, KM-H2, HDLM-2) compared to normal B-cell entities (*n* = 20: 5 × centroblasts (CB), 5 × centrocytes (CC), 5 × naive B-cells, 5 × memory B-cells).

For further filtering, we used the cumulative weighted context++ score (<−0.3) [23] from TargetScanHuman (http://www.targetscan.org/vert_72/), a database for target prediction. This threshold indicates relatively high probability of the respective miRNA-mRNA interaction. The filtering resulted in 20 genes for miR-148a-3p and three genes for miR-148a-5p. For further analysis, we have selected top nine genes for miR-148a-3p (*AKAP1, ARF4, CANX, CCT6A, C5orf30, HOMER1, IL15, FBN1, TFDP2*) and three genes for miR-148a-5p (*IFI6, SERPINH1, SUB1*).

**Figure 4:**
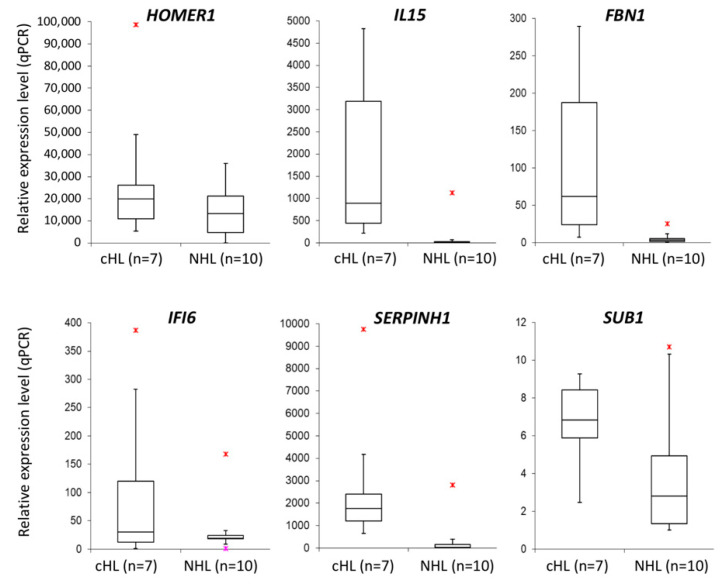
Overexpression of mir-148a putative target genes (*HOMER1, IL15, FBN1, IFI6, SERPINH1* and *SUB1*) in cHL cell lines (*n* = 7) as compared to NHL cell lines (*n* = 10) (real-time qPCR); 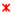
- max. outlier.

**Figure 5:**
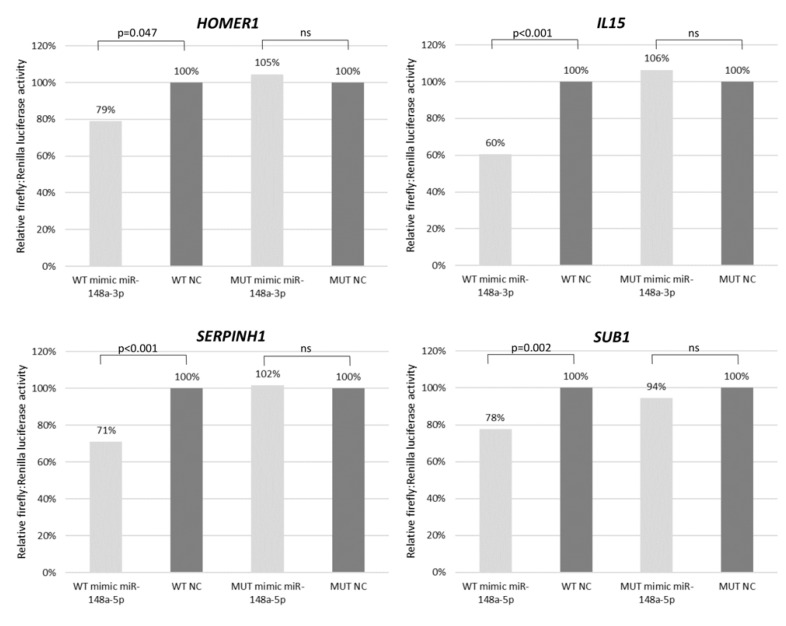
Interaction between mir-148a and its target genes (*HOMER, IL15, SERPINH1* and *SUB1* transcripts) shown by dual-luciferase reporter assay in HEK 293T cell line. WT–wild type 3′UTR, MUT–mutant 3′UTR with point mutations in miRNA binding site. Results presented as a mean from two independent experiment, each performed in three replications.

With the aim to validate the microarray expression data, we have performed real-time qPCR for selected genes in cHL cell lines (*n* = 7) and NHL cell lines (*n* = 10) as a control. Six candidate genes (*HOMER1* FC = 1.93, *IL15* FC = 11.87, *FBN1* FC = 17.24, *IFI6* FC = 2.50, *SERPINH1* FC = 6.26, *SUB1* FC = 1.78) were overexpressed at least 1.5-fold in cHL (Figure 4 and Supplementary Figure S1) and were further analyzed using dual-luciferase reporter assays. With this approach, we experimentally confirmed the interaction between miR-148a-3p and *IL15* (40% reduction, *p* < 0.01) and *HOMER1* (21% reduction, *p* = 0.047) transcripts. We have also demonstrated the interaction between miR-148a-5p and *SUB1* (22% reduction, *p* < 0.01) and *SERPINH1* (29% reduction, *p* < 0.01) transcripts (Figure 5).

Based on these results, we conclude that the loss of epigenetic repression of mir-148a in cHL contributes to the deregulation of several target genes which may alter important processes in cHL pathogenesis.

### 3.5. mir148a Overexpression Decreases Cell Proliferation

In order to put the observation of mir-148a downregulation in cHL in a functional context, we have established three cHL cell lines stably overexpressing mir-148a (KM-H2, L-540, L-1236).

**Figure 6:**
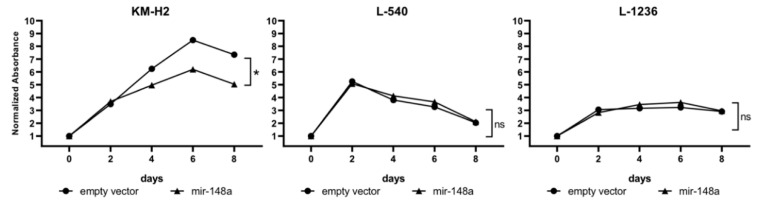
cHL cell lines (KM-H2, L-540, L-1236) viability after mir-148a overexpression (CCK-8 test). Results presented as a mean from three independent experiments, each performed in four replications.

By analyzing these cell lines using the CCK8 assay we have observed a significant decrease (*p* < 0.05) in cell proliferation after miR-148a overexpression in the KM-H2 cell line. On day 8 of the experiment a 32% reduction in cell proliferation was observed in the mir-148a-expressing cell line as compared to cells with the empty vector. miR-148a overexpression had no effect on the proliferation of L-1236 and L-540 cell lines (Figure 6 and Supplementary Figures S2–S4).

To analyze if the decrease in cell proliferation observed using the CCK8 test is related to differences in DNA replication, we conducted the Click-iT^®^ Plus EdU Alexa Fluor^®^ 647 Flow Cytometry Assay based on measurement of newly synthesized DNA in cells in S-phase. In line with the CCK8 test results, a decrease in DNA synthesis was observed for the KM-H2 cell line. On day 3 of culture, KM-H2 cells transduced with miR-148a expression vector showed a significantly lower percentage of cells with newly synthesized DNA as compared to cells transduced with the empty vector (35% vs. 49%, *p* = 0.03).

Taken together, functional studies performed in cell lines with induced mir-148a overexpression indicate that this microRNA has a negative influence on proliferation and DNA synthesis in a subset of cHL cell lines. Therefore, we assume that mir-148a may act as a tumor suppressor at least in some cHL cases.

## 4. Discussion

DNA methylation was previously shown to be an essential mechanism responsible for regulation of gene expression in the pathogenesis of cHL [3,4]. Our findings suggest that epigenetic silencing in cHL is not limited to protein coding genes but also plays an important role in deregulation of miRNA expression. miRNAs are responsible for fine-tuning the expression of protein coding genes involved in the maturation of B-cells, therefore deregulation of miRNA expression might contribute to the development of B-cell lymphomas [24,25].

As cHL is defined by a unique miRNA expression profile distinct from other B-cell lymphomas, one can expect the presence/absence of driver miRNAs having significant influence on cHL pathogenesis [26,27]. Following this lead, we have analyzed whether miRNAs found downregulated in cHL cell lines in our parallel study (our unpublished results, manuscript in preparation) have their promoter region located within or <1000 bp from a CpG island. We assumed that this is a strong indication that these miRNAs are epigenetically regulated and their DNA methylation level should be evaluated.

Consistent with our hypothesis, we have found promoter region hypermethylation of mir-339, mir-148a, mir-193a and mir-4488 in cHL cell lines. Moreover, with the exception of mir-4488, we have demonstrated that DNA methylation level inversely correlates with the expression level of these miRNAs. Importantly, mir-148a was found epigenetically attenuated exclusively in cHL.

Mir-148a was previously reported to be involved in the development of other type of cancers such as stomach, liver, lung, and breast cancer [28]. There are several ways in which it can contribute to cHL pathogenesis, however reports on its involvement in cHL are lacking so far. Firstly, mir-148a has been described as a component of a regulatory circuit that involves the NF-κB pathway, which is activated in HRS cells [29]. In this model, epigenetically mediated downregulation of mir-148a results in the overexpression of NF-κB in cancer cells. Secondly, by the interaction with methyltransferase DNMT3b and DNMT1, miR-148a-3p are directly involved in the process of DNA methylation, which is essential in the context of the global deregulation of methylation machinery in cHL [30,31]. Thirdly, mir-148a is expressed during physiological B-cell activation, and the promotor region of mir-148a is rich in motifs recognized by B-cell specific transcription factors like ELF1, EBF1, and E2A [9]. This is in line with our observation that this miRNA is unmethylated and expressed in GCB cells. Therefore, downregulation of mir-148a in cHL will likely contribute to the deregulation of the normal B-cell maturation process.

However, the exact function of most of the mir-148a target genes validated in our study in cHL remain poorly understood. Only the role of IL15, a prominent pro-inflammatory cytokine and important component of the growth and survival signals in cHL was previously described [32]. In the study by Ullrich et al., IL15 stimulation of cHL cell lines resulted in increased proliferation and activation of MAP kinase and JAK/STAT5 pathway. Interestingly, HOMER1 expression is also regulated via MAPK pathways and has a potential anti-apoptotic function [33]. SERPINH1 and SUB1 in turn were described as oncogenes in different cancer types promoting cell proliferation and invasion [34]. Exogenous overexpression of SUB1 in nude mice was shown to lead to transformation of normal multipotent fibroblast and tumorigenesis [35]. Taken together, the observed downregulation of mir-148a-3p/5p may lead to loss of transcriptional control of several cancer-related genes and at least partially explain their overexpression in cHL.

Lastly, in an attempt to understand the biological effect of the observed downregulation of mir-148a-3p/5p in cHL cell lines and microdissected HRS cells, we established three cHL cell lines (KM-H2, L-1236 and L-540) with stable mir-148a overexpression. Functional assays revealed the involvement of these miRNAs in negative regulation of proliferation in the KM-H2 cell line. We can only speculate that the composition of genetic alterations in the KM-H2 cell line makes it more sensitive to mir-148a overexpression than in the L-1236 and L-540 cell lines.

In summary, we identify mir-148a as a novel tumor-suppressive miRNA that is epigenetically inactivated in cHL.

## 5. Conclusions

We propose that miRNAs undergo epigenetic silencing by DNA hypermethylation in cHL in the same way as protein coding genes. Moreover, we identified mir-148a to be silenced by recurrent DNA hypermethylation which leads to loss of transcriptional control over several target genes including IL15 and HOMER1 and thereby contribute to cHL pathogenesis.

## Supplementary Materials

The following are available online at https://www.mdpi.com/2073-4409/9/10/2292/s1, Table S1: Cell culture conditions, Table S2: The clinical data of patients, Table S3: Primer sequences used in the study, Table S4: Assays used for bisulfite pyrosequencing (genomic localization based on GRCh37/hg19), Table S5: Oligonucleotide sequences used in luciferase assay. Figure S1: Expression of putative mir-148a target genes: *HOMER1*, *IL15*, *FBN1*, *IFI6*, *SERPINH1* and *SUB1* in seven cHL cell lines. Figure S2: KM-H2 viability after mir-148a overexpression (CCK-8 test). Results presented separately for each of the biological repetitions. Figure S3: L-540 viability after mir-148a overexpression (CCK-8 test). Results presented separately for each of the biological repetitions. Figure S4: L-1236 viability after mir-148a overexpression (CCK-8 test). Results presented separately for each of the biological repetitions.
