# Supplementary figures and images for: The Tumor Suppressive mir-148a Is Epigenetically Inactivated in Classical Hodgkin Lymphoma
Source: Cells. 2020 Oct 14;9(10):2292. doi: 10.3390/cells9102292 (PMC7602210; doi:10.3390/cells9102292)

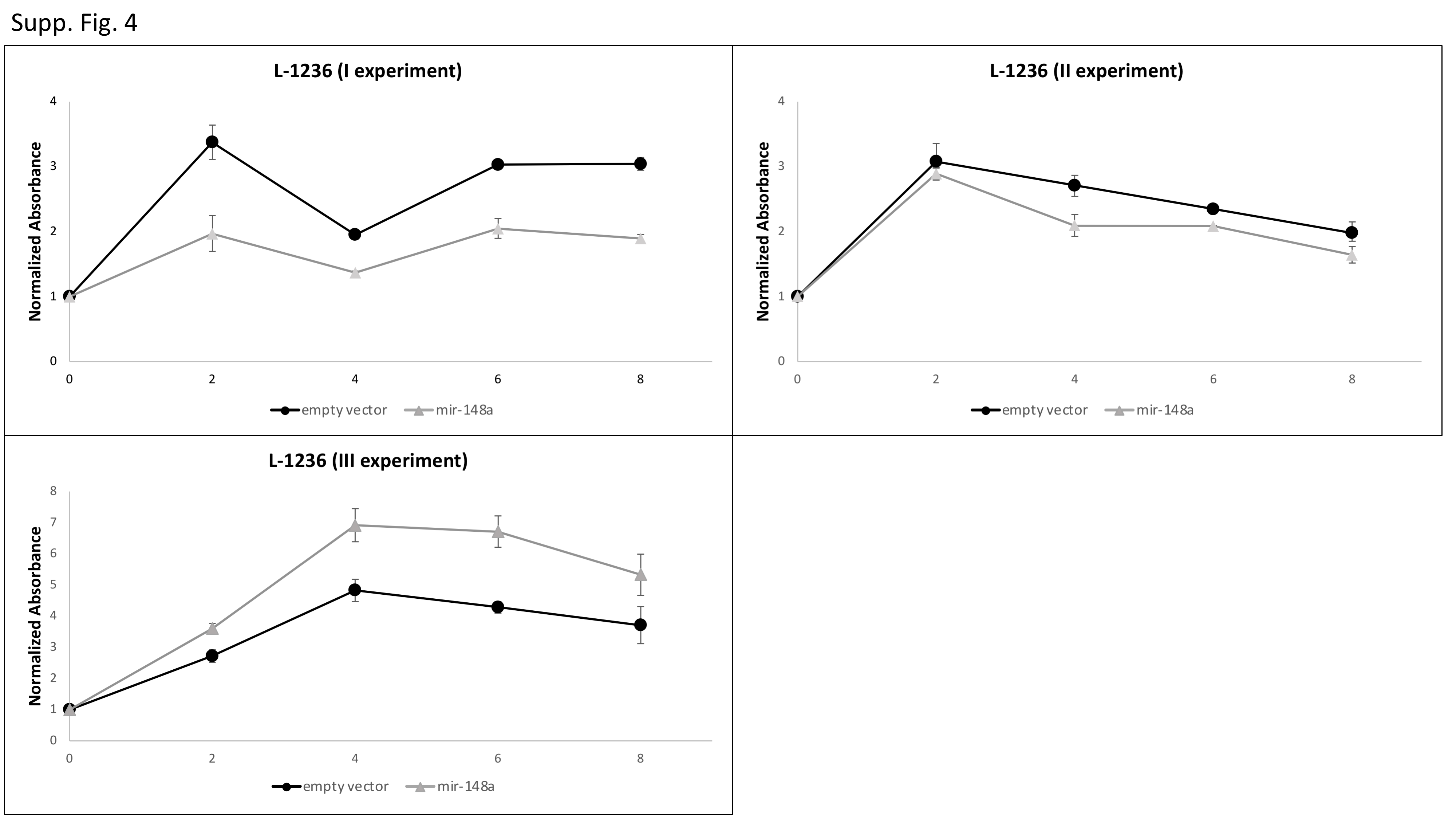

Supplement: Supplementary file 1 [file cells-09-02292-s001.zip › Supplementary figures/Supp. Fig. 4.tiff]

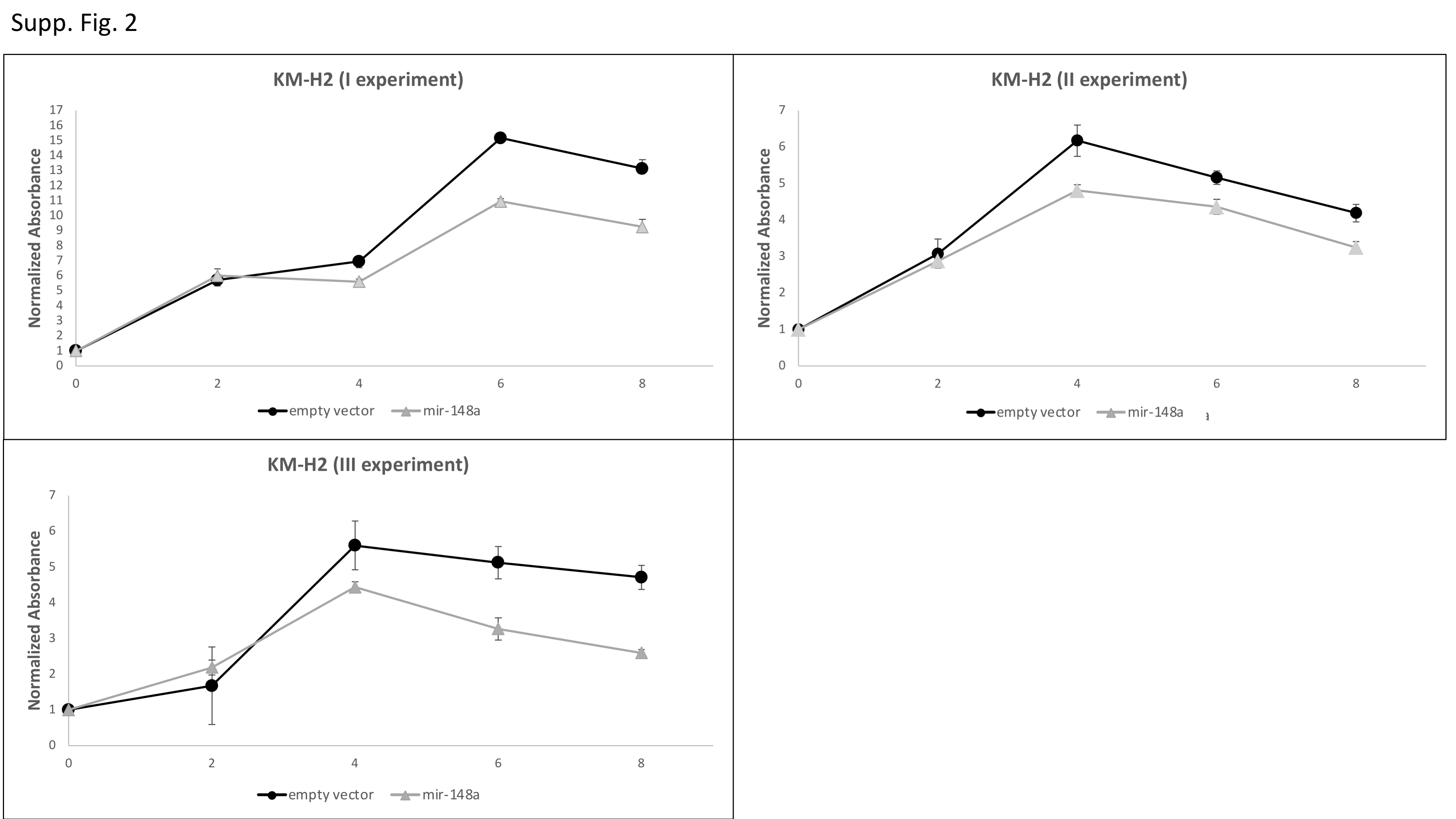

Supplement: Supplementary file 1 [file cells-09-02292-s001.zip › Supplementary figures/Supp. Fig. 2.tiff]

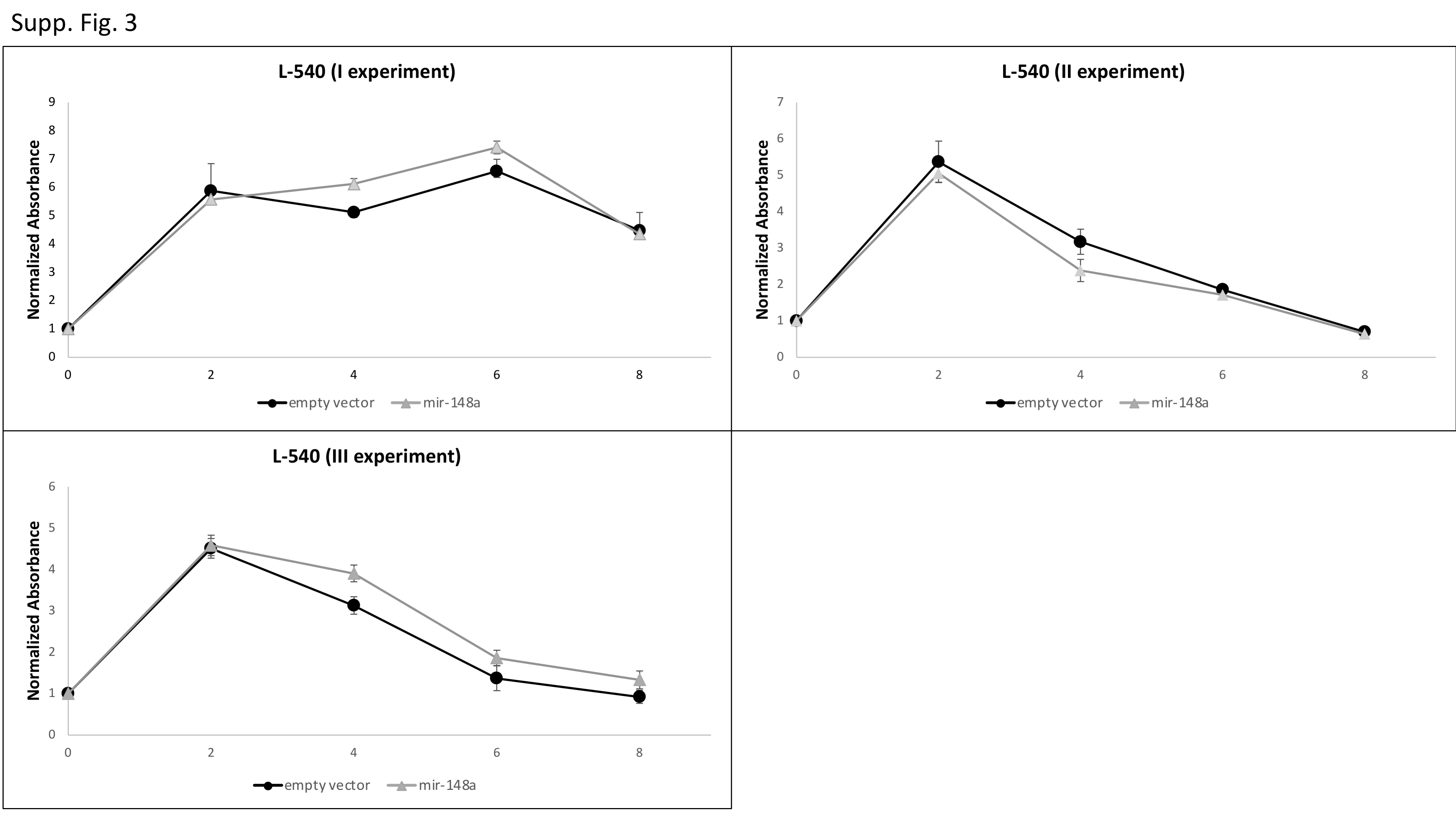

Supplement: Supplementary file 1 [file cells-09-02292-s001.zip › Supplementary figures/Supp. Fig. 3.tiff]

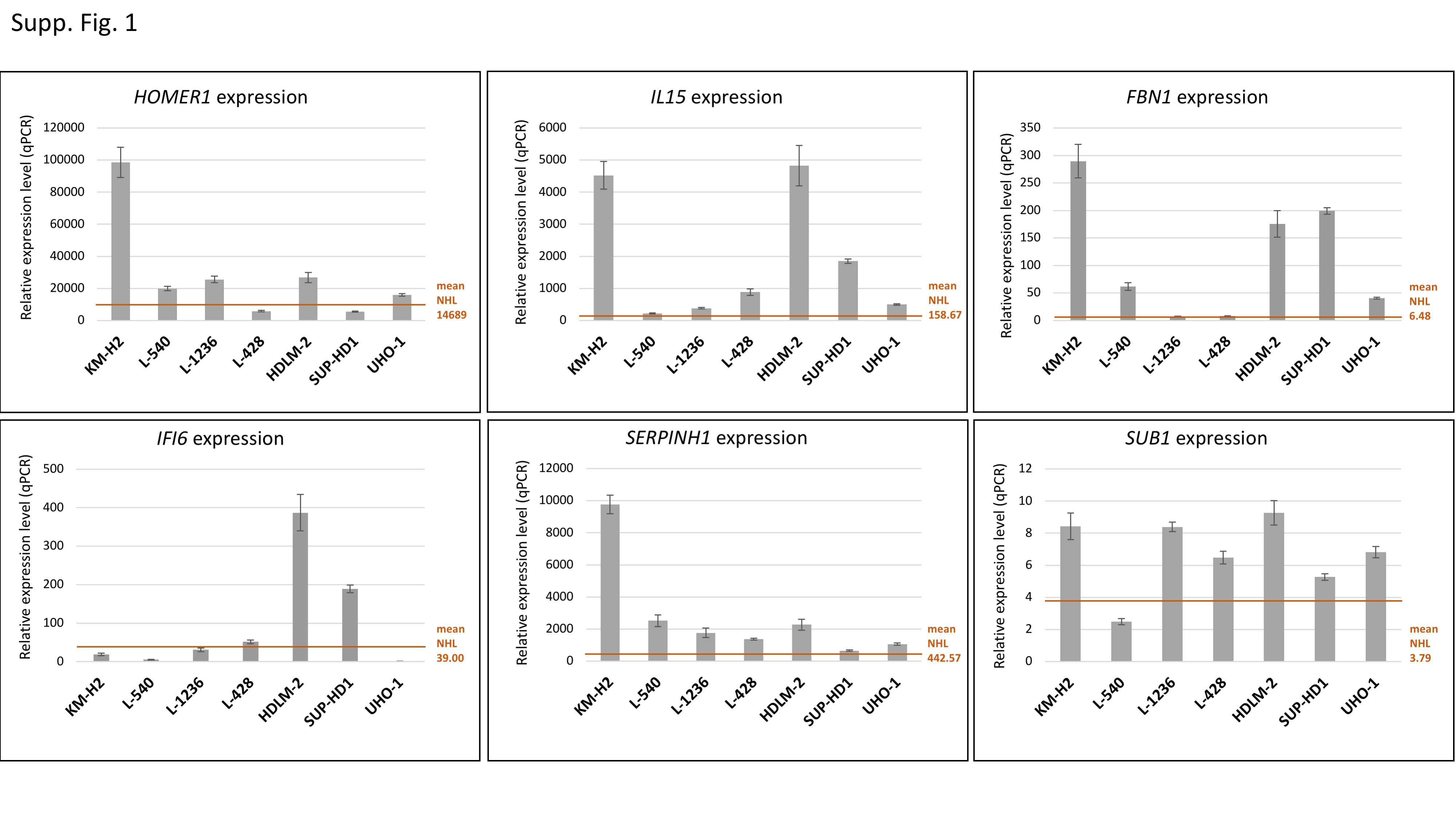

Supplement: Supplementary file 1 [file cells-09-02292-s001.zip › Supplementary figures/Supp. Fig. 1.tiff]
